# Visual SLAM for Dynamic Environments Based on Object Detection and Optical Flow for Dynamic Object Removal

**DOI:** 10.3390/s22197553

**Published:** 2022-10-05

**Authors:** Charalambos Theodorou, Vladan Velisavljevic, Vladimir Dyo

**Affiliations:** 1 School of Computer Science and Technology, University of Bedforshire, Luton LU1 3JU, UK; 2 Briteyellow Ltd., Bedford MK43 0BT, UK

**Keywords:** visual SLAM, object detection, simultaneous localization and mapping (SLAM)

## Abstract

In dynamic indoor environments and for a Visual Simultaneous Localization and Mapping (vSLAM) system to operate, moving objects should be considered because they could affect the system’s visual odometer stability and its position estimation accuracy. vSLAM can use feature points or a sequence of images, as it is the only source of input that can perform localization while simultaneously creating a map of the environment. A vSLAM system based on ORB-SLAM3 and on YOLOR was proposed in this paper. The newly proposed system in combination with an object detection model (YOLOX) applied on extracted feature points is capable of achieving 2–4% better accuracy compared to VPS-SLAM and DS-SLAM. Static feature points such as signs and benches were used to calculate the camera position, and dynamic moving objects were eliminated by using the tracking thread. A specific custom personal dataset that includes indoor and outdoor RGB-D pictures of train stations, including dynamic objects and high density of people, ground truth data, sequence data, and video recordings of the train stations and X, Y, Z data was used to validate and evaluate the proposed method. The results show that ORB-SLAM3 with YOLOR as object detection achieves 89.54% of accuracy in dynamic indoor environments compared to previous systems such as VPS-SLAM.

## 1. Introduction

Feature-based SLAM depends on feature points and keyframes in order to build a map of an unknown environment, and vSLAM can achieve robust performance without requiring any noticeable changes in a static or rigid environment [1]. Direct SLAM uses all camera pixels to resolve the world around the sensor(s) based on principles from photogrammetry. Instead of extracting pixels from the image and keeping them in 3D space, direct methods track their constrained aspects (colour, brightness, intensity gradient) over time [2]. Instead of geometric reprojection errors, this method minimizes photometric errors [3]. While both the feature-based and direct SLAM algorithms can distinguish feature types of stationary objects, neither of them can differentiate between different feature types in dynamic environments, such as train stations. These dynamic environments will produce data error associations when matching point pairs of dynamic feature points. Consequently, the visual odometer can no longer accurately estimate the pose of the object and the camera can no longer track the pose accurately. Recently, in order to overcome these difficulties, research on vSLAM in dynamic environments has received much attention [4].

Currently, there are few ways to detect dynamic moving objects in an unknown environment. The first method, which is based on geometry, is optical flow [5], which includes temporal redundancy [6] and the background subtraction method (BSM) [7]. This method works by analysing pixels in an image, which is more accurate for detecting moving objects, but has problems with high computational consumption and poor performance in real-time.

In recent years, many researchers have begun applying computer vision and deep learning technologies to vSLAM systems with the aim of creating semantic maps and removing moving objects. It is important to note that deep learning techniques have significant issues [8], but despite this, they are capable of greatly improving the performance of SLAM by detecting and removing dynamic objects [9]. Firstly, semantic segmentation networks such as R-CNN [10] are computationally expensive, making them impractical for real-time or robotic applications. A second problem is that neural networks can only detect objects that have been previously observed. Without prior knowledge, it is hard for the network to detect and identify what the object is. Therefore, pretraining the model is important. The third limitation is the lack of robustness in dynamic environments. If the environment is large, with high density of people and objects, the accuracy of the object detection model is low. As part of the vSLAM optimisation process, moving objects are either detected and then treated as outliers, or tracked separately [11]. Humans, for example, can be found in many real-life environments. Despite the fact that some dynamic objects can be viewed as noise, the vast majority violate the static environment assumption, so many existing methods of visual odometry are insufficient for real-world applications. The fourth limitation is that some vSLAM algorithms do not always work indoors, especially if the environment is large and the density of people is high. Train stations and airports are the most common environments that vSLAM algorithms tend to fail.

This paper tries to solve the problems of low accuracy and poor real-time performance of existing visual SLAM system ORB-SLAM3 [12] in dynamic environments. ORB-SLAM3 was used at the baseline for SLAM, YOLOX [13] and YOLOR [14] are combined as an object detection models. The experiments were carried out using a custom dataset that includes train station data. The overall system is specific for Monocular and Stereo-based vSLAM in indoor dynamic environments with high density of people and objects.

## 2. Related Work

### 2.1. Visual Planar Semantic SLAM—VPS-SLAM—Geometric

The size and shape of semantic objects can be estimated using planar surfaces and odometry applied to geometrical data representations. VPS-SLAM [15], is a visual planar semantic SLAM framework developed using a graph-based approach to utilise previous state-of-the-art visual internal and object detection algorithms.

However, the algorithm produces large errors in highly dynamic scenes. A dynamic moving object in an environment can be located and identified with the depth data of a sensor and the outer point data of a camera, according to [16]. There is only one limitation to this approach: if the data contains uncertainties and errors, the accuracy decreases.

Pixels can be tracked in real-time without having to calculate feature descriptors, and can be matched to features using an approached called optical flow. Previously, an optical flow algorithm was proposed by Lucas et al., which was used to detect motion consistency among dynamic feature points. Based on this method, the camera’s image changes over time as K(t) increases. If a pixel has simple coordinates, such as x and y, its grayscale value is K(x, y, t). Consider an image with a fixed point with horizontal and vertical coordinates x and y, which change over time, at different times in the image. The optical flow method predicts a 2D fixed point.

In a similar vein, [17] determining the difference between adjacent frames to detect moving objects, Sun et al. stated that the algorithm fails in real time [18]. Combining clustering information from a depth map and the depth map by itself, detecting dynamic moving objects in an environment can be achieved by using only geometry and the matching outer points from adjacent frames. The only limitation of this approach is that the algorithm is dependent on the pose transformations between adjacent frames, which causes the accuracy to decrease.

### 2.2. Deep-Learning-Based vSLAM

Nowadays, researchers use deep learning with SLAM algorithms to handle dynamic moving objects in indoor environments. DS-SLAM [19], which is based on the ORB-SLAM2 framework [20], was proposed by Cao Yu et al. that uses the SegNet [21] network for semantic detection in an environment. Feature point states are transformed by the RANSAC algorithm [22], which is used to calculate the inter-frame transformation matrix of the pole line geometry.

The basic matrix is calculated using all the feature points in the image. If there are too many error points in the image, the basic matrix will show serious deviations. The algorithm can be evaluated using the TUM dataset [23]. In a similar study conducted by Berta Bescos et al. [11], the ORB-SLAM2 algorithm combined with deep learning was used for the extraction of dynamic feature points in scenes. In a real-world environment, mask-RCNN is limited by the fact that it is not able to be used in real-time, resulting in poor results on the TUM dataset. Through a semantic mask derived from DUNet [24] and multi-view geometry, DDL-SLAM [25] detects dynamic objects, reconstructing the background obscured by dynamic objects using the image inpainting method. As pixel-level mask computation occurs when handling dynamic objects, this method also does not take place in real time.

A semantic SLAM system was proposed by Y. Fan et al. by utilising the BlitzNet [26] to estimate a movable object’s bounding box and mask. By using epipolar constraints, static matching points can be found in dynamic environments [27]. An epipolar constraint is a point in the environment that matches depth. Due to the lack of prior knowledge of the objects, this approach does not perform well, and it cannot work in real time.

## 3. System Overview

Next, we present an overview of the initial ORB-SLAM3 framework and its components. An improved ORB-SLAM3 is presented here, along with the object detection models YOLOX and YOLOR, as well as the prediction results.

### 3.1. ORB-SLAM3 Framework

For the first time, ORB-SLAM-VI presented a visual–inertial SLAM system that reuses the associated short-medium and long-term data as input to a visual–inertial BA based on preintegrated IMUs [28]. As a result of the slow initialization technique of the inertial measurement unit, the robustness and accuracy of the system were adversely affected.

To improve the initialization process of the system, researchers have proposed a solution that minimizes the 3D errors of points and not the reprojection errors in an environment. The noise in the IMU process is ignored and the bias, velocity, and feature depth [29] are retrieved.

**Figure 1:**
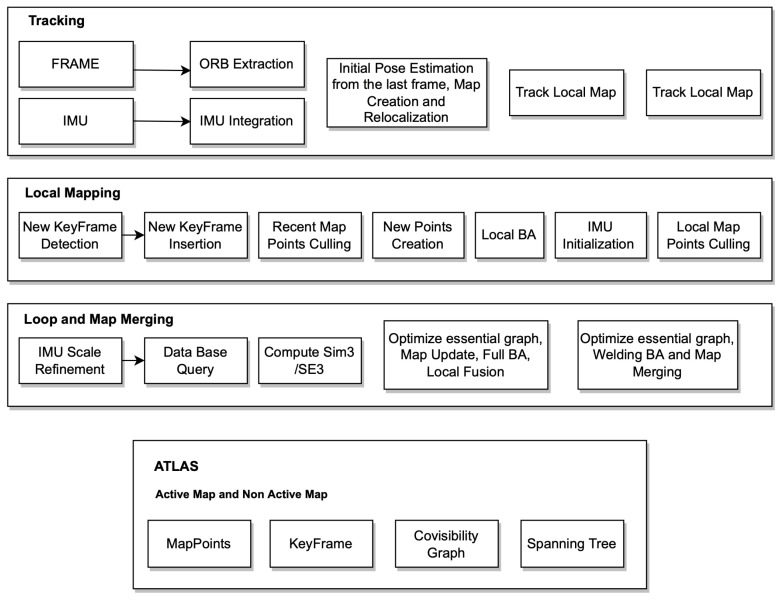
ORB-SLAM3 system architecture.

ORB-SLAM3 is built on top of ORB-SLAM2 and ORB-SLAM-VI. The system operates in visual or visual–inertial modes, using either monocular, stereo, or RGB-D sensors and a variety of cameras such as pinhole and fisheye cameras. The system architecture is shown in Figure 1.

1. **Tracking thread** is responsible for real-time processing of all sensor information and computing the position of the current frame in relation to the active map in order to minimize the reprojection error of the matched map features. Additionally, it determines whether the current frame becomes a keyframe. In visual–inertial mode, it is possible to estimate the inertial residuals from the optimisation to determine the body velocity and IMU biases. Tracking threads attempt to relocate the current frame across multiple Atlas maps when tracking is lost. Upon relocalisation, tracking is resumed, and the map is switched if necessary. After a certain period of time, if the active map is not stored, it will be replaced with a new active map, and the active map will no longer be active.
2. **Local mapping thread** is responsible for removing points that are not used and adding new points and keyframes to the map while improving it visually. MAP estimation is also used to initialize and refine the IMU parameters in the case of inertial sensors.
3. **Loop and map merging thread** is responsible for creating a single map by combining the two maps, and intently becomes the active one. A BA is then run in a separate thread to improve the map without affecting the real-time performance of the system.
4. **Atlas** is a collection of disconnected maps, and each one is represented by a separate map. The local mapping thread continuously optimises and grows the localised map, while new keyframes are added to the tracking thread and stored in the database. An inactive map in the Atlas is called a non-active map. A unique keyframe database is used to store all the maps, loop closing and to reload the map for relocalisation purposes.

### 3.2. Algorithm Framework

**Figure 2:**
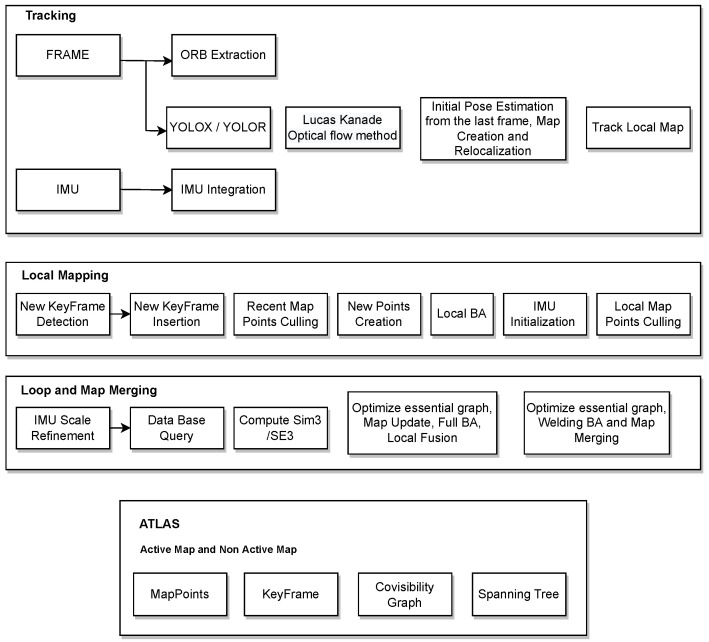
ORB-SLAM3 with YOLO system architecture.

ORB-SLAM3 suffers from reduced positioning accuracy and poor robustness in dynamic environments due to moving objects. There are four main modules in visual SLAM: visual odometer, back-end optimisation, loop detection, and mapping. The visual odometer mainly predicts the motion between images. The back-end optimisation optimises the prediction of the visual odometer to obtain relatively accurate transformations between image frames. Closed-loop detection detects whether the camera has been at the current position before. It can reduce the motion error by optimising the posture again through the back-end optimisation if it has already been in the current position. Based on the estimated camera positions, a map is constructed to describe the environment. A visual SLAM framework consists of the four modules listed above. To finalise the architecture of the framework, an objected detection model was used to detect objects as well as the optical flow method to check if the objects are moving and remove them. As a result, this paper introduces an object detection thread that is built on top of the original ORB-SLAM3 algorithm Figure 2 to detect moving objects. To extract feature points from the input images, the object detection models YOLOX and YOLOR are used. On the basis of the results and semantic data in an image, a module has been introduced that can remove dynamic objects.

If some objects are not detected or are detected and blurred, feature point matching may be necessary. Following the feature point matching process, the RANSAC algorithm [30] can be used to extract the essential matrix between two images. Finally, the dynamic removal process is carried out by using the LK optical flow method [31] and the pose between the adjusted frames is estimated with the remaining dynamic feature points.

### 3.3. YOLOR

**Figure 3:**
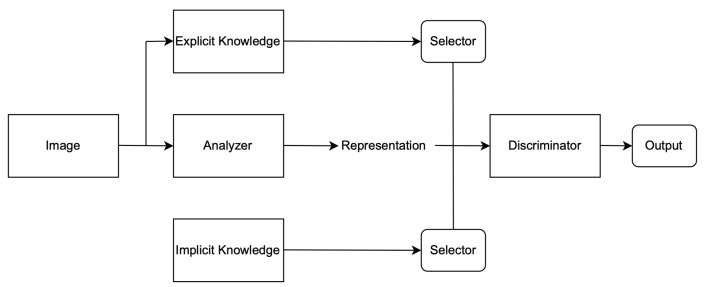
YOLOR system architecture.

YOLOR combines implicit and explicit knowledge to accomplish diverse tasks, such as learning a general representation. This representation can also be used to complete various tasks. With less than one in ten thousand additional parameters and computations, the accuracy of the model is improved. The implicit learning process of YOLOR is effective Figure 3 due to the fact that it uses multitask learning and not singular learning. it has a module that is responsible for refining the predictions and another module that is responsible for improving and making space on the kernel. A detailed explanation of YOLOR system architecture is described below:

1. **Explicit deep learning** can be carried out in multiple ways. One of them is Transformer, which uses query, key, or value to gain self-attention. Another way to obtain attention is through non-local networks, which mainly extract attention pairwise. Using input data to automatically select the appropriate kernel is another common method for explicit deep learning.
2. **Implicit deep learning** methods include implicit neural representations and deep equilibrium models. In the former, discrete inputs are converted into parameterized continuous mappings, while in the latter, implicit learning is converted into residual form neural networks and equilibrium points are calculated.

**Figure 4:**
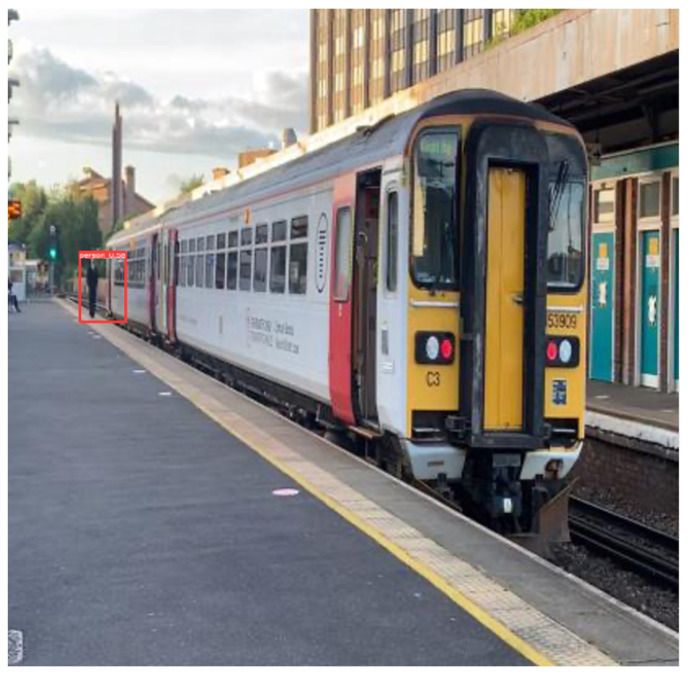
Detection results on YOLOR on custom dataset.

**Figure 5:**
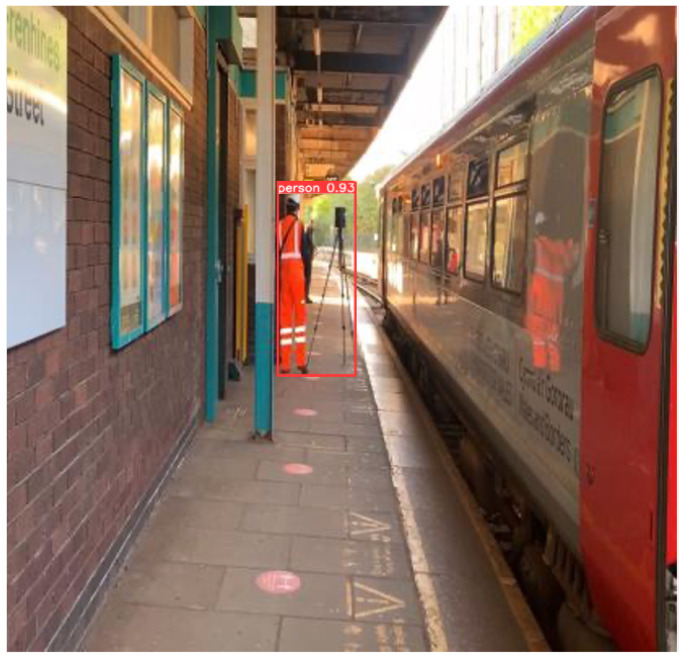
Detection results on YOLOR on custom dataset.

Implicit knowledge can be applied in a single model when it comes to improving the predictions for multi-task learning and preparing the model for object detection if the model takes as an input a whole image such as YOLOR. Finally, to improve and organise the layers in the model architecture (feature alignment), the hyperparameters were set to the default, the same as YOLOv4; the baseline of the model was YOLOv4—CSP [32]; and the Leaky ReLU activation function was used. On the MSCOCO dataset [33], 6.30% AP50 was obtained, making the model more robust and accurate over previous existing object detection models. For this study, YOLOR was used and tested on a custom dataset Figure 4 and Figure 5.

### 3.4. YOLOX

YOLOX achieves a significant challenging performance by using an anchor-free approach over existing object detection models such us YOLOv3, YOLOv4, and YOLO5. YOLOX runs on top of YOLOv3 with Darknet53 as baseline, and it uses a decoupled head, which is essential for YOLO. It is proven that it increases accuracy. It may still be beneficial to optimise those high-quality predictions in order to reduce the extreme imbalance caused by positive/negative sampling during training.

**Figure 6:**
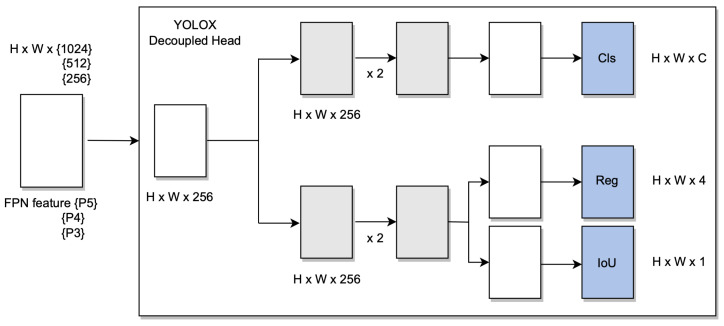
YOLOX system architecture.

In addition, to make the model respond to unexpected challenges, two new convolution layers, one-to-one label assignments, and stop gradients were created. The only limitation is that the performance of the model decreases. In addition to DarkNet53, YOLOX was tested on a custom dataset with different batch sizes, which showed significant improvement against unexpected challenges Figure 6.

**Figure 7:**
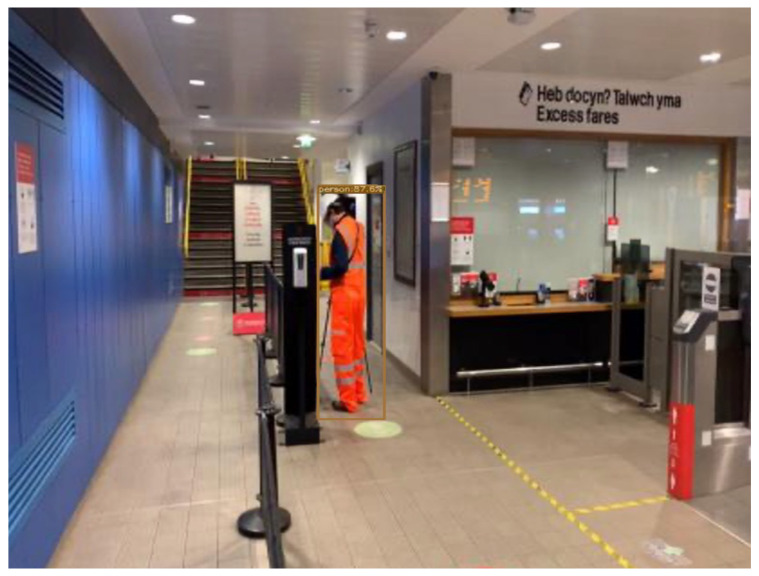
Object detection results of YOLOX on custom dataset.

**Figure 8:**
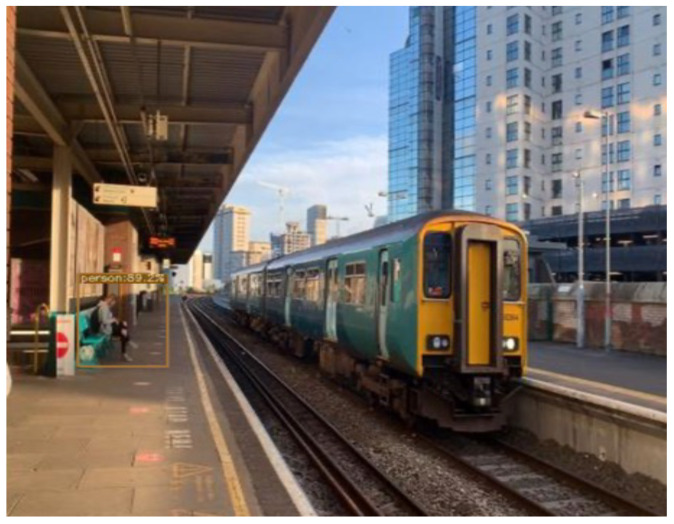
Object detection results of YOLOX on custom dataset.

The hyperparameters were set to default, and stochastic gradient descent (SGD) was used. YOLOX uses the same system architecture as DarkNet53 [34] and a pooling layer YOLOv3-SPP [35] that is used to remove fixed size constraints. On the MSCOCO dataset 65.4% AP50 was obtained with cosine learning rate, EMA added on the weights. Figure 7 and Figure 8 shows the object detection results of YOLOX on our custom dataset.

## 4. Dynamic Moving Object Removal

The apparent movement of an object, edge, or surface in a visual scene is the result of the relative motion of the viewer (eye or camera) and the scene. The LucasKanade method [36] is one of the most popular computer vision and optical flow estimation methods. To solve the basic optical flow equations for all pixels, the least squares criterion is used, and since there is no information about the flow in homogeneous regions of an image, it is a strictly local.

Bounding boxes for dynamic objects are generated during the object detection. Using prior knowledge to determine the dynamic feature points, the elimination process is as follows: The extracted feature points $X_{p}$ of an image are denoted when the i-th happens to be an input. With prior knowledge, $X_{p}$ = {$x_{1},x_{2},x_{3},\ldots x_{n}$} are all the dynamic feature points and can be expressed as $N_{p}$ when the input image is fed to the model. $N_{p}$ = {$n_{1},n_{2},n_{3},\ldots n_{n}$} is the prediction bounding box $N_{p}$. If $x_{p}$ belongs in $X_{p}$ (*j* = 1, 2, 3, …*n*) then it is considered as feature point and it is removed. The rest are denoted as $C_{p}$.

*Lucas Kanade Optical Flow Method*

If the brightness of the pixels in the image is the same and if the time difference between frames is short, the greyscale is constant, and it can be determined by:

(1) P(i,v,z)=P(i+di,v+dv,z+dz)

where *z*, *z* + *dz* represent the reciprocal times of adjacent frames, P(i,v,z) and P(i+di,v+dv,z+dz) represent the pixel points positions in the image. If there is only a small time interval between adjacent image frames, Taylor series expansion to the right of Equation (1) can be used to obtain:

(2) $$P\left(i+di,v+dv,z+dz\right)\approx P\left(i,v,z\right)+\frac{\partial P}{\partial x}\delta x+\frac{\partial P}{\partial y}\delta y+\frac{\partial P}{\partial t}\delta t$$

By combining Equations (1) and (2) by δt and dividing them:

(3) $$\frac{\partial P}{\partial x}\frac{\delta x}{\delta t}+\frac{\partial P}{\partial y}\frac{\delta y}{\delta t}=-\frac{\partial P}{\partial t}$$

$\frac{\delta x}{\delta t}$ and $\frac{\delta y}{\delta t}$ represent the velocity on the *x,y* axis and can be denoted as *z* and *v*. By denoting $\frac{\partial P}{\partial x}$ to $P_{i}$, $\frac{\partial P}{\partial y}$ to $P_{j}$ and the change of greyscale with time as $P_{f}$, Equation (3) can be rewritten more compactly in a matrix form as:

(4) $$\left[I_{i}I_{j}\right]\left[\begin{array}{c} z\\ v \end{array}\right]=-I_{f}$$

**Figure 9:**
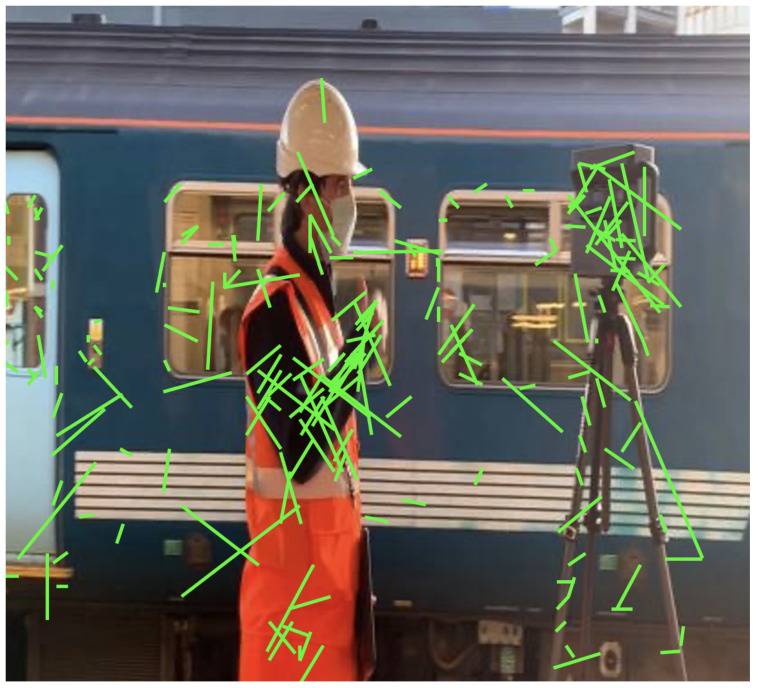
Experimental results of Lucas Kanade Optical flow method. Optical flow at high or normal wavelength is represented by the green line, while optical flow at low wavelength is represented by the green point.

After object detection, the optical flow size for static feature points is calculated. It is possible to determine whether a feature point is dynamic in terms of its mean value and standard deviation using Equations (5) and (6).

(5) $$|W_{i}-W_{avg}|>2W_{std}$$

(6) $$|W_{i}-W_{avg}|>W_{thr1}\left(W_{std}<W_{thr2}\right)$$

where $W_{i}$ represents the size of *i*-th feature point, $W_{avg},W_{std},W_{thr1}$ and $W_{thr2}$ are the mean, std, and the threshold values, respectively. From the experimental results of optical flow in Figure 9, the difference between dynamic and static feature points is shown.

## 5. Experiments and Results

### 5.1. Dataset

In this experiment, a custom dataset that includes RGB-D dynamic indoor images of train stations such as Birmingham, Cardiff Central, Cardiff Queen Street, Chester, Newport, Pontypridd, Shrewsbury, Jewellery Quarter, Smethwick Galton Bridge, The Hawthorns, Wolverhampton Bus station, and Wolverhampton train station, 360${}^{\circ }$ images, videos of the train stations (Outdoors and Indoors), *x, y, z* data, ground truth data and sequence data was constructed to evaluate the performance of the proposed algorithm.

The data were collected by the author for the purpose of several different projects of Briteyellow company. All the data were then preprocessed and merged together to create a custom dataset for this experiment that includes 16,139 images and 12 video recordings. The data were split into low and high dynamic scene data. The collection of the data was performed using high-quality 360${}^{\circ }$ cameras. The original size of the image data is 1920 × 1080, but it was later resized to 416 × 416. The video duration of each rail station is roughly 2 min and 15 s.

The specific model of the camera used was Ricoh Theta Z1. A mobile app was then used to process and export all the images. The equipment used for this experiment was a MacBook Pro with a 2.6 GHz 6-Core Intel Cor i7 processor, 32 GB RAM, Intel UHD graphics 630 1536 MB and Ubuntu 22.04 LTS as system environment running on Virtual Box.

### 5.2. Analysis

In this paper, the high dynamic scene data Fr2/xyz_walking, Fr2/rpy_walking, Fr2/train_station_walking, Fr2/signs_walking and low dynamic scene data Fr2/xyz_static, Fr2/rpy_static, Fr2/train_station_static, Fr2/signs_static were used for experimental verification. The evaluation metrics that was used is Absolute Pose Error (APE), Root Mean Square Error (RMSE), Median and Mean. For vSLAM systems, the absolute distance between the estimated and the ground truth trajectory is another important metric that can be used to assess the global consistency of the estimated trajectory. Due to the fact that both trajectories can be specified in any coordinate frame, they must be aligned first. RMSE reflects the difference between the real value and the observed value. It is important to observe the mean and median error values in order to determine the pose estimation accuracy. The proposed algorithm was compared with VPS-SLAM. The same proposed algorithm was tested with YOLOR instead of YOLOX using the same custom dataset.

**Figure 10:**
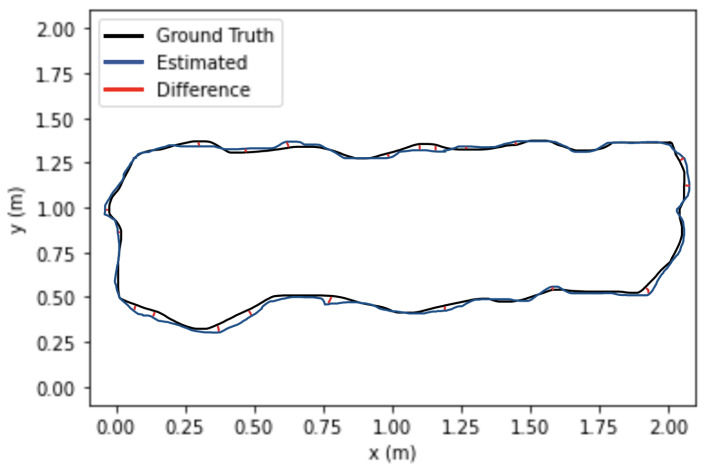
Absolute Trajectory Error (RMSE) of ORB-SLAM3 / YOLOR.

Figure 10 shows the trajectory comparison of the camera of ORB-SLAM3 compared to the proposed algorithm applied on xyz_walking and rpy_walking sequence from the proposed custom dataset. Compared to the actual trajectory of the camera, the trajectory estimated by the proposed algorithm is more accurate and closer to the actual trajectory.

**Figure 11:**
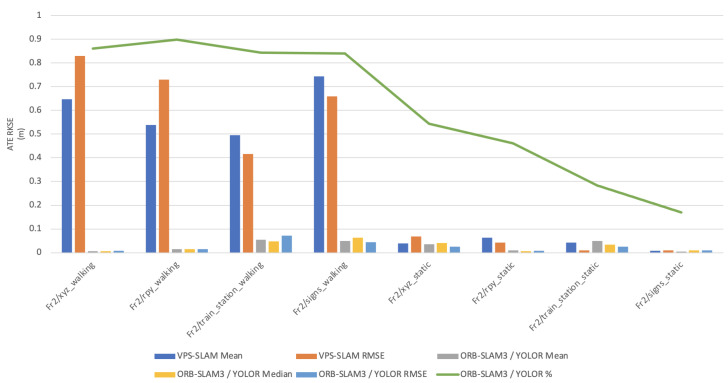
Absolute Trajectory Error (RMSE) distribution of VPS-SLAM and ORB-SLAM3/YOLOR.

**Figure 12:**
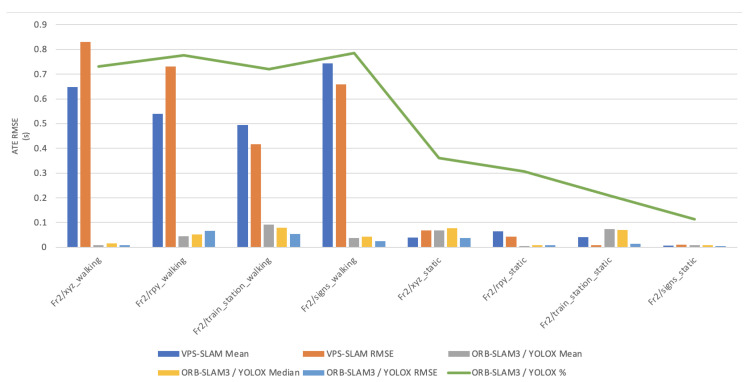
Absolute Trajectory Error (RMSE) distribution of VPS-SLAM and ORB-SLAM3/YOLOR.

**Figure 13:**
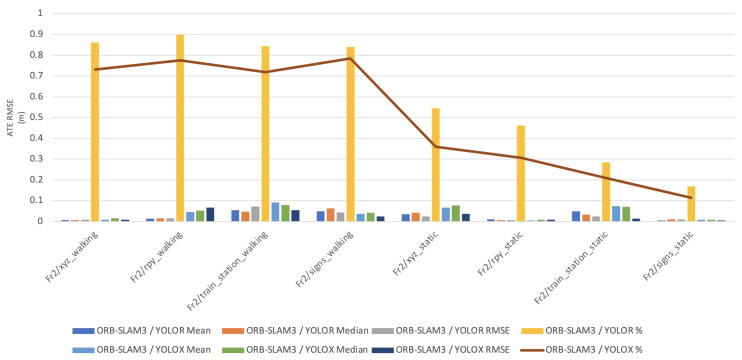
Absolute Trajectory Error (RMSE) distribution of ORB-SLAM3/YOLOR—ORB-SLAM3/YOLOX.

**Figure 14:**
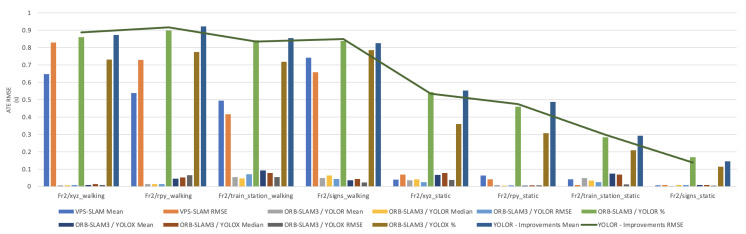
Overall Absolute Trajectory Error (RMSE) distribution.

**Figure 15:**
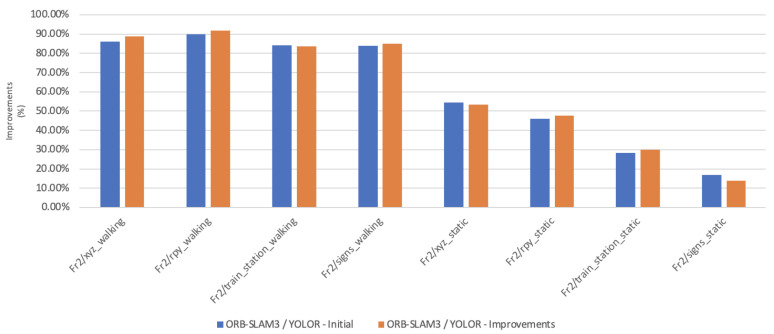
Accuracy improvements of ORB-SLAM3/YOLOR.

Figure 11, Figure 12, Figure 13, Figure 14 and Figure 15 shows how ORB-SLAM results are distributed in terms of errors combined with YOLOR and YOLOX when applied on xyz_walking.

Table 1 shows that in a complex dynamic environment with high density of people, the proposed algorithm achieved 88.76% improvement in the RMSE compared to ORB-SLAM3. Nevertheless, while experimenting in low dynamic environments VPS-SLAM achieved better results without interference from dynamic objects. The pose estimation interference problem caused by dynamic moving objects was overcome by combining object detection with ORB-SLAM3.

## 6. Discussion

Table 1 shows experimental results that indicate the track_station_walking dataset should not be regarded as improving. During the experimental phase, tracking failures occurred while using the Fr2_xyz_walking dataset. Based on the analysis of the dataset, we can see that a few images of people and signs are blurred. In a few instances, while using the Fr2_xyz_walking dataset, when the camera was moving, the people and objects in the images could not be detected by the network, which caused the accuracy to drop and the tracking to fail. As part of future research, object detection models could be optimised to improve accuracy and speed during this process, making it easier to detect dynamic objects and reducing their impact on vSLAM.

## 7. Conclusions

Moving objects can affect estimation accuracy in indoor dynamic environments, which can lead to tracking errors. The aim of this paper was to use an object detection model capable of handling moving objects in an indoor dynamic environment based on the recent ORB-SLAM3 framework. The YOLOX and YOLOR object detection algorithms have been tested to detect moving objects in an environment. For pose estimation, only static feature points were used. Dynamic point tracking was performed after static points were filtered out before tracking. Compared to VPS-SLAM, YOLOX combined with ORB-SLAM3 improved accuracy by 2–4% in highly dynamic indoor environments with pedestrians. This algorithm has certain advantages over other algorithms of the same type, both in terms of accuracy and performance.

## Figures and Tables

**Table 1:** YOLOX and YOLOR absolute pose error comparison with VPS-SLAM and ORB-SLAM3.

Sequences	VPS-SLAM		ORB-SLAM3/YOLOR				ORB-SLAM3/YOLOX				Improvements	
	Mean	RMSE	Mean	Median	RMSE	%	Mean	Median	RMSE	%	Mean	RMSE
Fr2/xyz_walking	0.6479	0.8298	0.0071	0.0065	0.0088	86.12%	0.0085	0.0153	0.0091	73.08%	87.43%	88.76%
Fr2/rpy_walking	0.5391	0.7302	0.0143	0.0151	0.0152	89.85%	0.0453	0.0521	0.0664	77.56%	92.31%	91.72%
Fr2/train_station_walking	0.4947	0.4169	0.0539	0.0480	0.0716	84.30%	0.0924	0.0785	0.0542	71.97%	85.47%	83.59%
Fr2/signs_walking	0.7428	0.6592	0.0489	0.0631	0.0441	83.97%	0.0369	0.0428	0.0241	78.55%	82.65%	84.96%
Fr2/xyz_static	0.0392	0.0684	0.0356	0.0414	0.0247	54.39%	0.067	0.0774	0.0377	36.02%	55.32%	53.44%
Fr2/rpy_static	0.0639	0.0425	0.0095	0.0063	0.0075	46.11%	0.0053	0.0080	0.0078	30.74%	48.76%	47.55%
Fr2/train_station_static	0.0418	0.0089	0.0495	0.0338	0.0247	28.42%	0.0743	0.0699	0.0136	20.87%	29.22%	29.85%
Fr2/signs_static	0.0073	0.0097	0.0044	0.0097	0.0098	16.96%	0.0082	0.0094	0.0057	11.41%	14.54%	13.78%

## Data Availability

Not applicable at the moment. The data cannot be publicly available at the moment because they are part of other company projects. The data will be publicly available after a couple of months because there are changes that need to be made on the company’s website. After that, the data will be publicly available for free to researchers at https://www.briteyellow.com accessed on 20 September 2022.
